# Breaking-up and breaking the norm: intergenerational divorce transmission among two ethnolinguistic groups

**DOI:** 10.1017/ehs.2025.9

**Published:** 2025-02-18

**Authors:** Caroline Uggla

**Affiliations:** Åbo Akademi University, Abo, VS, Finland

**Keywords:** divorce, separation, intergenerational transmission, ethnic minorities, Finland

## Abstract

Individuals who experience divorce in childhood are more likely to divorce themselves as adults. Notably, the magnitude of the intergenerational divorce transmission is stronger for groups among whom divorce is rare. This transmission may reflect differences in mating strategies passed from parent to child, or differences in cultural norms between groups. Sociologists and demographers have struggled to disentangle socioeconomic and cultural factors, because groups that are less wealthy also tend to have higher divorce rates. We use data from Finland, where two native ethnolinguistic groups with comparable socioeconomic characteristics – but different divorce risks – live side by side: Swedish-speakers and Finnish-speakers. Using register data on the entire Finnish population (*N* = 554,337 couples 1987–2020), we examine separation risk as a function of parental divorce. Data suggest that the intergenerational transmission is greater among Swedish-speakers, who have an overall lower separation rate. Group differences in separation risk persist even after controlling for socioeconomic factors and each partner’s experience of parental divorce. Notably, Finnish-speaking couples who reside in Swedish-dominated areas have both somewhat *lower* separation risk, and *higher* intergenerational transmission than their peers in Finnish-dominated areas. These results point to a cultural transmission of separation, beyond strong socioeconomic factors.

## Graphical abstract

**Figure:**
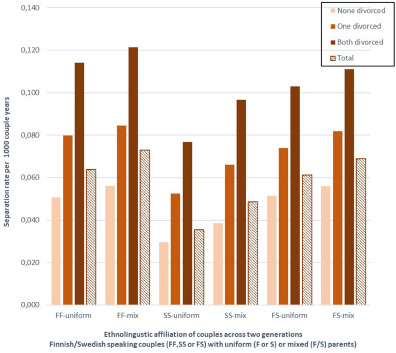

## Social media summary:

How far does the apple fall from the tree? Divorce transmission varies by frequency within the ethnic group in Finland.

## 1. Introduction

Children of divorced parents are more likely than others to divorce as adults. In the non-evolutionary sciences, understanding the mechanisms behind divorce is motivated by the fact that children from ‘intact’ families often fare better than those who experience divorce. Detrimental effects of parental divorce or separation are pervasive and have been documented across a wide range of societies. Among hunter-gatherers, such as the Ache of Paraguay, children whose parents divorce have lower survival than peers whose parents are still married (Hill & Hurtado, 1996, see also Blurton Jones et al., 2000). In post-industrialized societies a suite of negative effects for children of divorced parents have been documented, including impacts on school grades, lower enrolment in higher education, and higher likelihood of behavioural problems (see Amato, 2010 for review).

Although there is a consensus among sociologists and demographers that parental divorce negatively affects children in Europe and the US, effects of parental separation are not uniform between, or within, populations. Given that marriage practices are widely variable, it is not surprising that the effects of marital dissolution vary between groups. In some societies, marriages are informal and both partners can end a union without repercussions or social stigma. In others, such as many historical European societies, strict norms enforced long-term monogamy and divorce was only granted for specific reasons. In contemporary societies some mothers have access to close kin who can substitute any reduced investment from fathers upon divorce, whereas others do not. In welfare states such as the Nordic countries, single mothers can be buffered against economic costs of separation through financial support. When such support is not available, offspring may suffer from worsened socioeconomic conditions, and be more likely to get caught in a ‘cycle of divorce’.

One pattern of heterogeneity of divorce is particularly notable: intergenerational effects of divorce appear to be greater in groups where divorce or separation is rare (e.g. McLanahan & Sandefur, 1994). In an examination of 17 European countries and the US, the intergenerational transmission of divorce was greater in countries where the proportion of women who had experienced parental divorce was higher (Dronkers & Harkonen, 2008). The intergenerational transmission of separation/divorce also differs *within* countries. In the US, Black people are more likely to dissolve pairbonds than Whites, yet the effect of divorce on offspring is greater among Whites (Heard, 2007). In the Netherlands, individuals of Caribbean origin have high separation and low intergenerational transmission, whereas White ancestral Dutch individuals have low divorce and high intergenerational transmission of that behaviour (Kalmijn, 2010). In Sweden, the extent to which children are affected by parent’s separation differ between different immigrant groups. In groups where parental separation is common, for example migrants of Chilean origin, the decrease in children’s school grades is smaller than among children of parents of ethnic groups where separation is rare, for example mothers of Bosnian origin (Erman & Härkönen, 2017).

Two main explanations have been put forward by non-evolutionary social scientists to explain why parental divorce or separation is likely to be perpetuated by the next generation. The first concerns resource access: low socioeconomic status is associated with separation, and parental separation often leads to a reduction in resources. Demographic traits that correlate with lower socioeconomic status may also play a part here; individuals who grow up poor are more likely to enter partnerships at younger ages, and a young age at partnership entry is in itself associated with a higher separation risk (Amato & Patterson, 2017). This pattern is consistent with evolutionary life history theory, which states that individuals with fewer resources may incur higher fitness by speeding up life events such as maturation and reproduction (Stearns, 1992).

The second explanation is that children who have grown up in environments where many couples split up will be more likely to divorce or separate themselves because it is *culturally accepted* to do so. This type of argument made by sociologists is analogous to the evolutionary perceptive of cultural norms. Once divorce is common within a group and more socially accepted, individuals do not suffer severe social sanctions when breaking up with a mate. These individuals may have a lower threshold to end a relationship that does not live up to the expectations.

There is much evidence to support the notion that divorce is socially transmitted. In Europe, North America and much of the post-industrialized world, divorce rates increased during the twentieth century, and especially after the 1960s and 1970s with changing ideals about family life. The increased divorce rate coincided with increased labour market participation of women, but the pace of the increase cannot be explained by women’s economic independence alone. Divorce norms differ not only over time, but also cross-sectionally between sub-groups. For instance, child immigrants in the United States with origins in low-divorce countries in Europe are less likely to divorce if they reside close to a sizeable number of co-ethnics (Furtado, Marcén & Sevilla, 2013), even when socioeconomic status and home-country factors such as gross domestic product (GDP) and religiosity are accounted for. In social network analysis, divorce has been shown to spread among friends in the US (Mcdermott, Fowler, Christakis & Mcdermott, 2013). Changes to policy and legislation can also bring about normative shifts in divorce attitudes. When Denmark removed the mandatory 6-month waiting period for uncontested divorces in 2013, there was a short-term high spike as couples were allowed to divorce earlier, but also a long-term effect. The Danish divorce rate stabilized at a 10% higher level compared to before the reform (Fallesen, 2021). Thus, although separation is closely related to socioeconomic factors, it may also be affected by rapidly changing cultural norms.

Evolutionary scientists have long recognized that teasing apart ecological explanations from cultural mechanisms is a sticky business (Mace, 2014). It is plausible that *both* the transmission of low socioeconomic status and cultural norms may contribute to the intergenerational transmission of divorce. Intergenerational transmission of socioeconomic resources may also help explain the inverse frequency-dependent transmission, that is, that offspring are impacted more by parental divorce when divorce is rare. Sociologists have argued that the relative disadvantage after separation may be lower in groups where parents have few resources, simply because such children begin at a lower level. With regard to cultural norms, it is possible that children who live in a context where separation is common may suffer less stigma and thereby less negative impact. Thus, when divorce is common, the event itself may infer fewer behavioural problems, relatively more support, and better offspring outcomes than for those who experience parental separation when it is socially sanctioned.

In this paper, we use data from contemporary Finland and apply an evolutionary perspective to understand both why separation risk varies across subgroups, and why the magnitude of the intergenerational transmission of separation differs between such groups. Studies on the intergenerational effect of separation are mostly based on groups that differ both in their divorce norms and simultaneously have large socioeconomic differences. This is problematic because when these factors correlate strongly, one cannot disentangle reasons why separation may be transmitted from parents to offspring. Robustly accounting for socioeconomic and demographic factors that covary with separation is crucial to understand the intergenerational transmission. Many factors that are predictive of separation are more common among couples with low socioeconomic status, for example entering a pairbond at younger ages, living in unmarried cohabitation rather than in a marriage, and having lower education (Lyngstad & Jalovaara, 2010).

### 1.1. Study population

We use data on contemporary Finland to gain insight into the intergenerational transmission of separation. In contemporary Finland, two native ethnolinguistic groups live side by side: Finnish-speakers (95% of the population) and Swedish-speakers (5% of the population). In the population register, a person can have only one registered mother tongue. The ethnolinguistic division has profound impact on society, through separate social and cultural institutions, parallel school systems, geographic residential segregation, and even a separate Swedish-speaking army brigade (McRae, 1997). The two groups function like separate ethnicities in how they are traditionally defined (cf. Gordon, 1964; Saarela, Kolk & Uggla, 2022). Finnish and Swedish do not share recent linguistic roots, and are not intuitively understandable to each other, like Swedish, Norwegian and Danish are. Swedish- and Finnish-speakers in contemporary Finland are comparable in terms of socioeconomic and most demographic characteristics (Saarela, 2021). Finland was part of Sweden until 1809, a Grand Duchy under Russian rule until 1917, and has thereafter been a republic with two official languages, Finnish and Swedish, and the two groups have equal constitutional rights.

Howevber, Swedish and Finnish-speakers do differ markedly in one regard: the stability of their pairbonds. Marriages and cohabiting relationships consisting of individuals from the Swedish-speaking minority have lower separation risk than unions between two Finnish-speaking partners (Saarela & Finnäs, 2014). Comparisons to the other Nordic countries, which have similar family demography, reveal that it is the Swedish-speaking low separation rate that is striking, whereas the Finnish-speaking couples’ is on par with other comparable contexts (Saarela & Finnäs, 2018). Endogamous Finnish marriages have about twice as high separation risk as endogamous Swedish ones (Finnäs, 1997). Exogamous Finnish–Swedish unions have an even higher divorce risk, about 10% higher than that of endogamous Finnish unions. However, Saarela and Finnäs (2014) have shown that when considering cohabiting unions only (i.e. not marriages), the risk of separation is only marginally stronger among exogamous Swedish–Finnish-speaking unions than endogamous Finnish-speaking couples (endogamous Swedish-speaking couples are still at lower levels). High levels of social integration and low mobility of Swedish-speakers have been proposed as mechanisms behind the stability of endogamous Swedish unions, although these arguments have not been empirically verified (Finnäs, 1997; Saarela & Finnäs, 2018).

Since the 1950s, when an increasing number of Finnish-speakers moved into the Swedish-speaking settlement area of Finland, the Swedish-speaking population has been facing demographic changes. At this time, approximately 20% of the Swedish-speaking population married a Finnish-speaking spouse (Finnäs, 1986, 2012). This figure rose gradually until the 1980s when it levelled off. Today about 40% the unions of Swedish-speakers are to a Finnish-speaker (Saarela, 2021). In the national registers, a person can be registered with only one mother tongue (chosen by parents shortly after the birth). With an increase in the number of unions across the ethnolinguistic border during the twentieth century, a substantial number of children are raised by parents from both ethnolinguistic groups, and about two-thirds of the children in mixed Finnish–Swedish families are registered as Swedish-speakers.

Finland is characterized by comparatively high gender equality as found in the Nordic countries. Both men and women and initiate divorce and women are typically active in the labour market and have their own income. Living together as a couple without being married is common in the Nordic countries, including Finland. Around half of all children are born to non-married cohabiting parents (Andersson, Jalovaara, Uggla & Saarela, 2022). The lines between non-marital cohabitations and marriages are blurred, as most long-term unions eventually turn into marriages.

As ethnolinguistically exogamous unions are the most labile (Finnäs, 1997; Saarela & Finnäs, 2014), individuals from both ethnolinguistic groups may pay some cost to partnering outside of their own group. The group differences cannot be explained by socioeconomic, religious or demographic factors (Saarela et al., 2022), but the extent to which they depend on experience of parental divorce is not known. One focal aim of this paper is thus to examine whether the Finnish–Swedish divorce/separation gradient in Finland can be related to differences in divorce experiences in childhood.

### 1.2. Contribution and research questions

Our paper contributes to the study of the intergenerational transmission of separation in four important ways. First, as outlined above, the main aim of this paper is to examine the intergenerational transmission of separation among two distinct two ethnolinguistic groups who share similar socioeconomic and demographic factors. Second, we are unique in examining the transmission of separation as a function of the residential context, that is, contrasting couples in Finnish-speaking and Swedish-speaking areas, all else equal. In Finland, some areas are strong holds of Swedish-speakers. Swedish-speaking Finns, who live on the west and south coastline, often come from families that lived in the same municipalities for generations and in areas where the separation rate is relatively low (Monti & Saarela, 2023; Saarela & Finnäs, 2018). Drawing on this fact, we are able to test whether the intergenerational transmission of separation is associated with the separation norm of the residential area.

Third, we include data on divorce (and death) of *both sets of parents* to the focal couple. This is important because the separation risk at the couple level is influenced by parents on both sides. Yet including both partners’ parental unions is rare in the literature, which often has used data only on one individual and her/his parents (an exception is Kailaheimo-Lönnqvist, Fasang, Jalovaara and Struffolino (2021) from Finland, Storksen, Røysamb, Gjessing, Moum and Tambs (2007) for Norway and Wolfinger (2003) for the USA). We are thus able to examine whether there is a dose-dependent relationship between parental separation and own separation, whether the focal couple is more likely to dissolve if both parties had experienced divorce, than if one or none had.

Fourth, we use register data to capture *all cohabiting unions, irrespective of whether they are marital or non-marital*, from age 18 and onwards, for the entire population of Finland. Our data span more than 30 years, from 1987 to 2020. Most previous studies are from a US context and have covered only marriages, which may bias result towards unions that are more stable and consist of individuals with higher access to resources than individuals who live in cohabitating unions. To examine cohabitations is particularly important in this Nordic context. In Finland, non-marital cohabitations are by far the most common form of pairbond. During our study period, only 6.7% of all new unions started out as marital unions.

We address two main analytical questions:

1. Can differences in separation risk between Swedish- and Finnish-speakers in Finland be explained by differences in parental divorce?
2. Is the magnitude of the intergenerational separation transmission greater for (a) Swedish-speakers, among whom separation is rare, and (b) in geographical areas where separation rate is low?

### 1.3. Ethnolinguistic difference in separation risk and the role of parental divorce

Previous evidence from Finland suggests that Swedish-speakers have lower divorce rates even when controlling for socioeconomic factors. This *may* point to differences in cultural norms regarding partnerships between the two ethnic groups. For instance, the low mobility of Swedish-speakers may be associated with high social cohesion that might work as a protection against divorce. A difference in divorce norm could explain why the stability of Swedish endogamous couples is especially high in Western Finland (where many Swedish-speakers reside) compared to Southern Finland (which is more ethnolinguistically mixed) (Saarela & Finnäs, 2018). Saarela and Finnäs (2018) also found that the lower divorce risk of Swedish-speakers is constant over union/marital duration, but the reasons for this pattern is not known. Notably, the role of divorce in the parental generation has not hereto been comprehensively analysed. This is an important omission, because group differences in separation may disappear once the experience of parental divorce of both partners is controlled for. If so, it would support the hypothesis that parental behaviour or its demographic or socioeconomic correlates, mostly explain differences between ethnic groups. If, on the other hand, there are sizeable ethnolinguistic differences in separation risk *even after* controlling for parental behaviour and sociodemographic characteristics, this implies some residual differences that may be due to cultural norms. Given that we have ethnolinguistic data across two generations, it is possible to make predictions about whether ego’s own or their parents’ ethnolinguistic affiliation is a stronger predictor. However, in previous research on partner choice both mattered (Uggla & Saarela, 2024), and so here we merely hypothesize that a higher degree of Swedish-speaking will be associated with a lower separation risk. This reasoning leads to our first hypothesis.

*Hypothesis 1: Swedish-speakers (SS-uniform and SS-mixed) have lower separation risk than Finnish-speakers (FF-uniform and FF-mixed), even after controlling for parental divorce and sociodemographic factors.*

### 1.4. Intergenerational transmission

Second, we examine the magnitude of the intergenerational transmission of separation. Based on previous evidence, the effect of parental separation on children’s subsequent separation will be stronger in groups among which divorce is rare (e.g. among Swedish-speakers) than where it is more common (e.g. among Finnish-speakers and exogamous FS couples). This leads to our second hypothesis.

*Hypothesis 2: The magnitude of the intergenerational transmission is lower for groups that have higher overall separation rate.*

### 1.5. Intergenerational transmission by local area

Third, we consider separation norms by area of residence. In Finland, some areas along the western and southern coastline are strongholds of Swedish-speakers. Migration from these areas is low, especially for Swedish-speaking families, many of whom have lived here for generations (Monti & Saarela, 2023). We draw on this fact to test whether couples who reside in areas where a majority are Swedish-speakers have (a) lower risk of separation, even if they are Finnish-speakers themselves, and (b) have higher intergenerational transmission of separation in these areas where separation is rare.

To our knowledge, this is the first study to explore whether the intergenerational transmission varies by geographically defined cultural areas. If demographic or socioeconomic factors explain differences between areas, this may be due to differences in resources or biases in who lives in such an area. If, on the other hand, couples who reside in predominantly Swedish-speaking areas are less likely to separate than those in predominantly Finnish-speaking areas even net of control variables, this may indicate that they respond to the cultural norm of the residential area. This leads to our third hypothesis.

*Hypothesis 3: Couples who reside in low-separation (Swedish-speaking) areas have lower separation risk than those who reside in high-separation (Finnish-speaking) areas, regardless of ethnolinguistic affiliation.*

Lastly, we test the intergenerational separation transmission by area: transmission should be stronger in magnitude in Swedish-dominated areas than in Finnish-dominated ones.

*Hypothesis 4: Couples who reside in low-separation (Swedish-speaking) areas have higher intergenerational transmission of separation than those who reside high-separation areas, regardless of ethnolinguistic affiliation.*

## 2. Methods

We use a collection of linked national population-wide registers from Finland. Finnish registers are world class and uniquely cover data on ethnolinguistic information through each person’s unique mother tongue, as well as records on both non-marital and marital cohabitation unions. Each person can be linked to their mother and father, as long as the parent was alive in 1970. Through anonymized person numbers we can link individuals to various socioeconomic variables and demographic controls, and to cohabitation by the residential address. The data are accessed through Statistics Finland’s FIONA system, and used with permission number TK-53-1370-17.

### 2.1. Cohabitation

Cohabitation (i.e. a co-residential partnership) is widespread in Finland, and a large part of such unions subsequently turn into marital unions (Andersson et al., 2022). Finland is one of the few countries in the world where cohabiting unions, regardless of whether the couple has children, can be identified in the population registers. Cohabitations are based on a definition by Statistics Finland that notes if a person is domiciled with an opposite-sex person, who is not a sibling or a parent, in the same dwelling beyond 90 days, and the age difference to the other person does not exceed 20 years. Cohabitation is also recognized irrespective of whether the couple has a common child. The cohabitation measure applied has been established as accurate (Lyngstad & Jalovaara, 2010), and conforms to international standards for the classification and identification of couples in households (Kennedy & Fitch, 2012).

The cohabitation data date back to 1987. Time at risk of a partnership starts at age 18, and we observe union entries in 1987–2020. We cover all individuals who have complete partnership histories by including unions where both the man and the woman were born 1970–1986. 1970 is the lower limit because cohabitation data start in 1987 (so those born 1970 are aged 18 then). 1986 is the upper limit to ensure that all individuals were born during the ‘marriage era’, not the cohabitation era that followed. All entries into (heterosexual) unions are considered, that is, second- or higher-order unions are also included and union order is controlled for. However, given how entangled non-marital cohabitation and marriage are, we refrain from analysing these two union forms separately. In Finland, many couples who have lived in cohabitations do marry eventually, and it is not unusual for marriage to occur after many years of living together. In fact, to fully distinguish cohabitations from marriages (as might be motivated in other cultural settings, e.g. the US) is not necessarily conceptually possible or desirable in Finland (see e.g. Andersson et al., 2022; Saarela & Finnäs, 2018). Nevertheless, we recognize that unions that start out as marital may differ in some ways, and therefore we do control for marital status at union entry.

### 2.2. Separation

The focal couple’s separation is based on whether they move apart, meaning that they are registered as not living together anymore, irrespective of whether they are unmarried or married. Separation occurs the year the couple is no longer registered at the same address, irrespective of whether they may divorce later or earlier than so. Parental separation refers to divorce from marital unions only, because data on non-marital cohabitation do not exist before 1987. This is not a major shortcoming, however, because unmarried cohabitation was not very common in Finland before the mid-1980s, and most couples with children eventually married. The parental divorce measure is based on both sets of parents of the focal couple, measured when each individual was 17 years old (i.e. time fixed). We code couples into the following categories: (a) both’s parents’ marriages were intact, (b) one’s parents’ marriage was intact, (c) no one’s parents’ marriage was intact, and (d) at least one parent had died. If a person has parents that divorced when they were, for example, 10 years old, and a parent died after that, they are coded as having experienced divorce. The death category is important to include since death is one way by which a marriage can be dissolved. However, given that this category is both empirically thorny and does not relate to our research question theoretically, it is not presented in the main results (see Appendix for full tables and estimates of all covariates).

### 2.3. Ethnolinguistic categories

Each person in Finland is registered with a unique mother tongue, that is, Finnish, Swedish, or any other. We study only those with Finnish or Swedish as their mother tongue, who amount to 86% of all persons in the study cohorts (immigrants are thus not included unless they also speak these languages). Among these, only 1% has a parent with other mother tongue than Finnish or Swedish, and they are excluded from analysis. For each couple, we thus know the woman’s, man’s and each of the four parents’ mother tongue. Following previous research (Uggla & Saarela, 2024), we use six categories to reflect the ethnolinguistic composition of the couple: (1) endogamous Finnish couples with uniform Finnish background, (2) endogamous Finnish couples with some mixed background, (3) endogamous Swedish couples with uniform Swedish background, (4) endogamous Swedish couples with some mixed background, (5) exogamous (F/S) couples with no mixed background (one person has two Swedish-speaking parents, and the other partner has two Finnish-speaking parents), (6) exogamous (F/S) couples with some mixed background (at least one of the parental union is mixed F/S). We refer to these six categories as: FF-uniform, FF-mixed, SS-mixed, SS-uniform, FS-uniform S/F and FS-mixed. See Table A1 for descriptive statistics of each group.

### 2.4. Control variables

We adjust for a number of control variables that may impact the couple’s separation risk. They are woman’s age at union entry, age difference of the couple, calendar year of union entry, marital status at union entry, union order of the woman and the man, number of children at entry, woman’s educational level at entry, man’s educational level at entry, woman’s religion at entry, man’s religion at entry, woman’s parents’ education, man’s parents’ education, woman’s full- and half-siblings, man’s full- and half-siblings (in order to capture differences in family composition that may impact own relationship duration), population density of municipality of residence at union entry, and proportion Swedish-speakers of municipality of residence at union entry. They are all categorized and described in more detail in Table A1.

### 2.5. Modelling strategy

Couples are followed until separation, death, emigration, or end of 2020, whichever comes first, and then become right-censored. The total number of couples is 554,337, and the total number of separations is 309,676. To examine differences in separation between ethnolinguistic groups and by the experience of parental divorce, we calculate separation rates, that is, number of divorces by couple years under risk for each category. To evaluate the differential impact of parental divorce across ethnolinguistic groups, we run discrete-time Cox regressions that include covariates. Area-level effects are evaluated by running separate regressions for municipalities that have at least 50% Swedish-speakers and those that have less Swedish-speaking population than that.

## 3. Results

### 3.1. Descriptive separation rates

**Figure 1.**
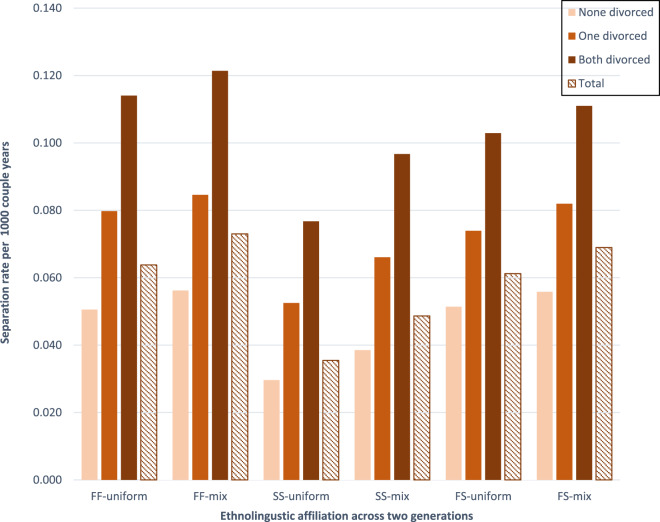
Risk of separation (by couple years at risk), across ethnolinguistic group (for the focal individuals in the couple and both sets of parents) and parental divorce. Both divorced: both the male and females’ parents had divorced before the year the focal individual turned 17. One divorced: one of the parental unions ended in divorced. None divorced: none of the individuals in the focal couple experienced parental divorced.

Figure 1 shows the separation rate (by couple years) across couples’ ethnolinguistic identity (across two generations) and parental divorce. The data show clear differences by parental divorce: couples where both individuals had parents whose marriages ended in divorce had a higher separation risk than couples where only one parental union had ended in divorce before the year ego turned 18 years. The highest rates are found among couples for whom both parental unions had ended in divorce.

There is an ethnolinguistic gradient in separation rate; Finnish-speaking endogamous couples with mixed background have the highest separation rate, followed by Finnish-speaking endogamous couples where both sets of parents have Finnish-speaking background (64–73 per 1000 years at risk, respectively). Thereafter Swedish-speaking endogamous with mixed background, and Swedish-speaking endogamous with uniform background have separation risks of 35–49 per 1000 years at risk, respectively. Exogamous F/S couples are intermediate between Swedish-speaking and Finnish-speaking couples with separation rates of 61–69 per 1000 years, depending on whether they have uniform or mixed background.

Table 1 shows results from Cox models: only a small part of the differences between FF, SS, and mixed FS couples in separation hazards can be attributed to parental divorce differences between these groups. This offers support for *Hypothesis 1*, that the higher likelihood of separation among Finnish-speakers remains after adjusting for the fact that Finnish-speakers are more likely to have experienced divorce in their own families during childhood or adolescence. The difference in separation hazards between Swedish-speaking couples where both sets of parents are Swedish-speakers, and Finnish-speaking couples with two sets of Finnish-speaking parents (the reference category) is reduced only slightly: 39% lower hazard become 35% lower hazard once experience of parental divorce is controlled for. After adjusting for socioeconomic and demographic controls (model 3), there are still large differences between the ethnolinguistic groups; SS couples with uniform Swedish backgrounds have 28% lower hazard of separation than FF endogamous couples. In the final model, also controlling for population density and share of Swedish-speakers in the municipality, the SS couples with uniform Swedish background still have 22% lower separation hazard compared to Finnish-speaking couples with uniform Finnish background (HR 0.78, 95% CI 0.75–0.81).

**Table 1.** Separation risk of couples based on ethnolinguistic identity (hazard ratios of separation with 95% confidence intervals). Model 1 contains only the couple’s ethnolinguistic identity, model 2 adds controls for whether the couple’s parents had separated (one set or both sets of parents), model 3 adds controls for socioeconomic and demographic variables. The last two models add geographical area controls: model 4 adds population density, and model 5 adds the percentage of Swedish-speakers at (county ‘kommun’) level

	Model 1	Model 2	Model 3	Model 4	Model 5
Couples’ ethnolinguistic affiliation (+ parents’)	HR 95% CIs	HR 95% CIs	HR 95% CIs	HR 95% CIs	HR 95% CIs
FF (uniform F)	1	1	1	1	1
FF (mixed F/S)	1.10(1.08,1.13)	1.06(1.03,1.08)	1.03(1.01,1.05)	1.02(1.00,1.04)	1.02(0.99,1.04)
SS (uniform S)	0.61(0.59,0.63)	0.65(0.63,0.67)	0.72(0.70,0.75)	0.75(0.73,0.77)	0.78(0.75,0.81)
SS (mixed F/S)	0.79(0.76,0.83)	0.82(0.78,0.85)	0.89(0.85,0.92)	0.91(0.87,0.94)	0.93(0.89,0.97)
F/S (uniform F/S)	0.94(0.92,0.97)	0.96(0.93,0.98)	1.02(0.99,1.05)	1.02(0.99,1.05)	1.02(0.99,1.05)
F/S (mixed F/S)	1.05(1.03,1.07)	1.03(1.02,1.05)	1.03(1.01,1.05)	1.02(1.00,1.04)	1.02(1.00,1.04)

Moreover, the results point to the importance of ethnolinguistic identity across *two generations*. Couples where both individuals are Swedish-speaking (SS) but at least one of the them has exogamous parents (i.e. one Finnish-speaking and one Swedish-speaking), have a higher separation risk than Swedish-speaking couples were both sets of parents are Swedish-speakers (i.e. uniform Swedish background). Finnish-speaking couples (FF) with mixed background had 10% higher hazards in Model 1 compared to FF with uniform Finnish background, but this difference attenuated when control variables were added. It seems that the ethnolinguistic affiliation of the couple themselves is more predictive of separation than the parental affiliation, a point to which we return in the discussion. Exogamous FS couples do not show significant differences in their separation hazards compared to Finnish-speakers in the full models.

**Figure 2.**
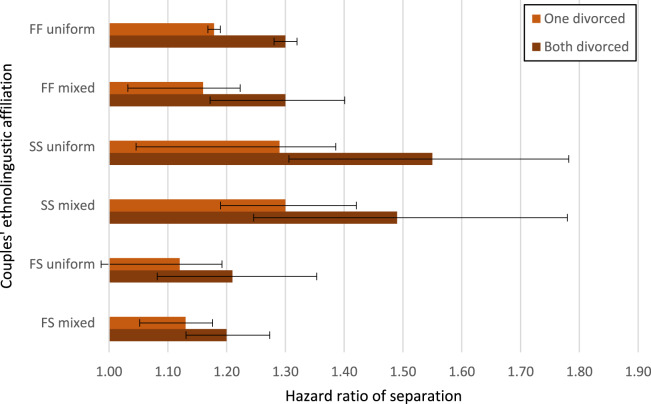
Separation risk by ethnicity and couple’s experience of parental divorce. Hazard ratios with 95% confidence intervals. Reference category: None divorced.

Next, we test *Hypothesis 2*, whether intergenerational transmission is greater among groups where separation is lower. Figure 2 displays hazard ratios (with 95% confidence intervals) for experience of parental divorce from the Cox proportional hazard models. The reference category is couples where none of the focal individuals had experienced parental divorce by the year they turned 17 years. The models control for all socioeconomic and demographic covariates of model 5 (see above). All results displayed come from the same model, only the reference categories have been changed. Broad confidence intervals are expected as some groups are small in absolute numbers. However, given that our data are based on the entire Finnish population, results can be interpreted without confidence intervals.

The overall levels of separation by ethnolinguistic groups are the following (as found in Fig. 1), lowest to highest SS-uniform < SS-mixed < FF-uniform < FF-mixed. Exogamous couples FS-uniform and FS-mixed fall in between, but we focus on endogamous couples here (either SS or FF) as it is more difficult to say both empirically and theoretically how norms among exogamous couples would be expressed.

The effect sizes of parental separation are greatest among the Swedish-speaking couples with uniform S backgrounds, and lower among Finnish-speaking and exogamous FS couples. Swedish-speaking couples where two partner had experienced parental separation had 50% higher hazard of separation compared to having two intact parental unions. If both of the parental unions dissolved, the focal couple has about 30% higher hazard of separation than Swedish-speakers with two intact parental unions. Comparable hazards in Finnish couples with endogamous background are around 30% and 15% for two and one parental unions ending in separation, respectively.

Exogamous F/S couples had the smallest separation hazards of parental divorce, but fall in between SS and FF in absolute separation risk. Thus, these results are overall in line with *Hypothesis 2*, that associations between parental divorce and one’s own separation are related to the frequency of separations within the ethnic group. There is not a perfect inverse correlation between separation rate and magnitude of intergenerational transmission when FS unions are considered. However, if only examining the four categories of Swedish-speaking and Finnish-speaking couples, the predicted pattern holds.

Furthermore, these data show that parental divorce positively predicts separation/divorce in the next generation. Because we have data on both sets of parents, we can conclude that the association between parental divorce and the focal couple’s separation is dose-dependent; couples where both individuals experienced parental divorce have higher separation hazards than couples where only one had experienced parental divorce. The lowest separation risk is found among couples where none had experienced separation.

### 3.2. Separation rates by residential area

Lastly, we turn to examine separation risk and the role of parental separation across different geographical areas. Swedish-speakers reside in certain regions, and we hypothesized that Swedish-speaking regions produce a low-separation norm – also for the Finnish-speakers who reside there (*Hypothesis 3*). Note that here we use three ethnolinguistic categories based on one generation (not two generations as above) for ease of interpretation and to have enough couples of each type within each area.

**Figure 3.**
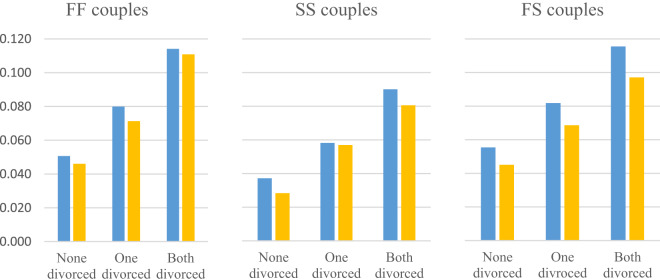
Separation rates by ethnolinguistic group of the couple (both Swedish (SS), both Finnish (FF), or mixed Finnish–Swedish (FS) residing in Swedish-dominated areas (yellow bars) where Swedish-speakers are 50% or more in Finnish-dominated areas (blue bars) where Finnish-speakers are 50% or more.

Figure 3 shows that the absolute rates of separation are higher in predominantly Finnish-speaking areas. This is true for all ethnicities, and across different categories of parental separation.

We then checked whether the differences in separation rates between Finnish/Swedish-dominated areas hold when adjusting for all sociodemographic confounders included in our analyses. In these adjusted models, Swedish-speakers in predominantly Swedish-speaking areas do still have 14% lower hazards of separation compared to a similar couple in a Finnish-speaking area HR 0.86 (95% CIs 0.81–0.94) (if none had divorced parents, which was the reference category). However, for Finnish-speakers, differences in separation risk across areas are just short of statistical significance when adjusting for socioeconomic and demographic characteristics, 0.91 (0.82, 1.01).

**Figure 4.**
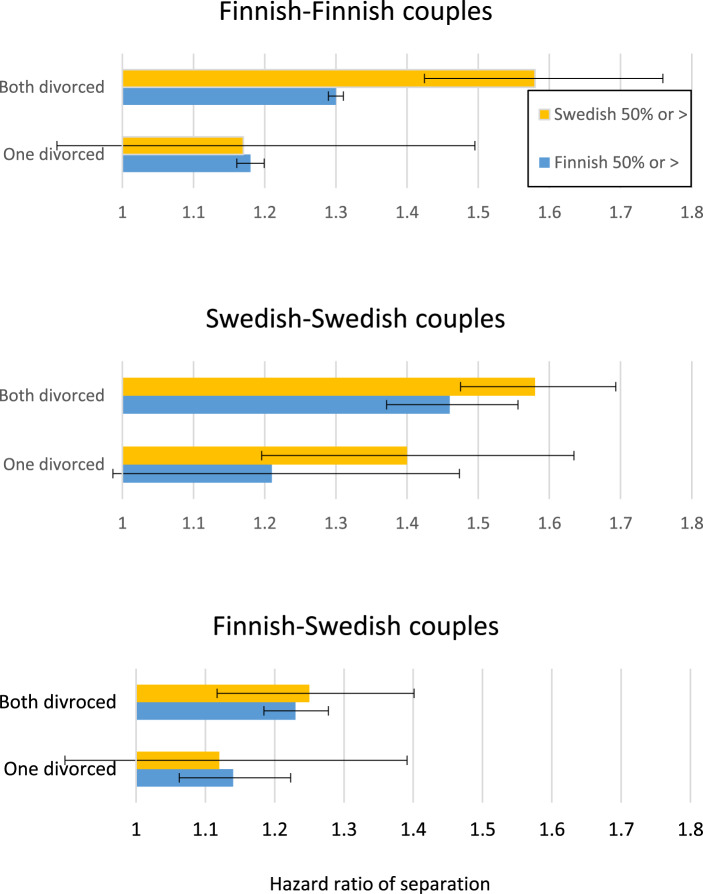
Hazard ratio of separation as a function of parental divorce (reference: none of the parents divorced), and areas of residence (Swedish-dominated area: Swedish-speakers 50% or more in municipality, or Finnish-dominated area (Finnish-speakers 50% or more).

Our final research question is whether couples in low separation areas have higher intergenerational transmission of separation – regardless of their ethnolinguistic affiliation. (*Hypothesis 4*). Figure 4a–c displays the magnitude of intergenerational transmission across areas. Swedish-speaking and Finnish-speaking couples with two sets of divorced parents (compared to two intact sets of parents) have higher hazards of separation if they reside in a Swedish-dominated area rather than in a Finnish one. Among Swedish-speaking couples, the effect size of parental separation is 1.58 in Swedish-dominated areas versus 1.46 in Finnish-dominated areas if ‘both separated’. Among Finnish-speaking couples hazard ratios were 1.58 in Swedish areas but only 1.30 in Finnish areas. However, there is no statistical difference in the ‘One divorced’ category across areas, and among exogamous FS couples the results do not differ across Swedish versus Finnish-speaking areas.

## 4. Discussion

We have examined the intergenerational transmission of separation among two native ethnolinguistic groups in Finland. Because the Swedish-speakers and Finnish-speakers are comparable in terms of socioeconomic and demographics, they present a rare opportunity to disentangle common observable antecedents of separation from cultural norms. Drawing on the unique data of complete cohabitation histories of the entire Finnish population, we contribute to the literature on separation risk across ethnic groups, and to the understanding of transmission of mating behaviour from parent to offspring.

These results are in line with previous evidence that Swedish-speakers have lower separation risk than Finnish-speakers. But over and above the individual’s ethnolinguistic affiliation we show that ethnolinguistic affiliation of the parents matter. Separation risk is lower for Swedish-speaking couples with uniform Swedish background, than Swedish-speaking couples with mixed backgrounds (where at least one of the four parents were registered as a Finnish-speaker). Similarly, Finnish-speaking couples with at least one Swedish parent have lower separation risk than Finnish-speaking couples where all four parents are Finnish-speakers.

### 4.1. Intergenerational transmission of separation

Intergenerational effects of separation were strong and dose-dependent. Couples where both sets of parents had divorced were far more likely to separate than couples where only one set of parents had divorced. This corroborates previous results from Finland, demonstrating an additive effect when both sets of parents were divorced (Kailaheimo-Lönnqvist et al., 2021). Multiplicative effects have been found in for instance the US and Norway, yet studies that are able to examine divorce behaviour of both sets of parents remain scarce. Kailaheimo-Lönnqvist et al. (2021) argued that in contemporary Finland having divorced parents implies a lowering of the threshold for separation among both of the individuals in the couple, rather than a spiralling of conflict that might be the case in settings where multiplicative effects of two sets of separated parents on offspring behaviour.

Despite strong intergenerational transmission, we found support for our first hypothesis that lower separation risk among Swedish-speakers persisted even after controlling for experience of parental divorce among both partners in the focal couples. That effects were attenuated when adjusting for education, age at marriage and other demographic variables, is not surprising. Yet, the residual differences were rather large and imply that other factors than standard demographic factors account for these differences.

Second, we studied the heterogeneity in the magnitude of intergenerational transmission of separation. Transmission from parent to offspring was, as hypothesised, highest among Swedish-speaking couples, and lowest among Finish-speaking and exogamous Finnish-Swedish (FS) couples. Overall this is in line with Hypothesis 2, but there was no perfect inverse correlation to absolute separation rates for respective ethnolinguistic group. For example, exogamous FS couples were situated in between in absolute separation risk, but had the longer intergenerational effects. Results for exogamous FS couples are somewhat tricky to interpret as these couples, by definition, is a blend of two communities, and may not have their own ‘community’.

### 4.2. Area differences in separation risk and its intergenerational transmission

In the analyses of residential areas it was interesting to note that, in line with *Hypothesis 3*, all ethnolinguistic groups had slightly higher separation rates in Finnish-speaking areas. This might imply that there is a separation norm that spills over to Swedish-speakers in Finnish areas. On the flip side, we also saw that Finnish couples who resided in Swedish areas, had lower separation rates than peers in Finnish areas. However, we do not claim that these patterns are causal. It is possible that Swedish individuals who do not wish to be part of the Swedish community, or who do not share values and attitudes of that community, are more prone to move to Finnish areas, and/or large urban regions, or vice versa.

The hazard of separation is greater if the couple both have divorced parents, and this effect is amplified in Swedish-dominated areas when few others in the residential area share the experience of having divorced parents. It is interesting that this area difference is seen not only among Swedish-speakers, but also among couples where both individuals speak Finnish. Overall the Finnish-speakers in Swedish areas are less likely to divorce and this could indicate that Finnish-speakers in the Swedish-speaking adopt the low divorce norm prevailing among Swedish-speakers. These models control for many demographic and socioeconomic factors that could confound the patterns, nor is it explained by population density.

Swedish-dominated communities are characterized by low residential mobility, which makes for a tight-knit community where norms can be upheld, and the benefits of group membership are strong (Monti & Saarela, 2023). Individuals in these communities may perceive the value of staying in a pairbond as higher than Finnish-speakers do and might suffer sanctions if they do break up. This is also consistent with the observation that couples who stay in rural areas have lower divorce risk (Finnäs, 1997). Similar arguments of the protective role of tight-knit communities have been made to explain the comparatively low divorce rate among Jews in the US (Glenn & Supancic, 1984).

It is important to note that the number of municipalities where more than 50% are registered as Swedish-speakers are much fewer than the other way around. In addition, the areas classified as Finnish-speaking here are very heterogeneous. Finnish areas comprise areas where almost all inhabitants are Finnish-speakers, as well as the Helsinki region where there are relatively more Swedish-speakers in total, along with ‘pockets’ of Swedish communities. Thus, Swedish areas and Finnish areas are not fully symmetrical.

Another possible explanation for the residual difference in separation behaviour is that it is not the social sanctions but the social support afforded to Swedish-speakers that depress separation risk. In other words, it may not be that Swedish-speakers have a ‘low divorce norm’ in their community, but also the fact that the Swedish-speakers *are* a community. In minority communities, social support networks may be more active and pertinent. A couple who are struggling may avoid separation by having friends and family nearby. The low residential mobility of Swedish-speakers may be a prerequisite for exerting norms, but could equally protect against separation as it means social relations can more easily be maintained. The stability of social networks could be a potential explanation for why also the Finnish-speakers in Swedish-speaking areas had somewhat lower risk of separation.

It would be odd to discuss the mating strategies in contemporary Finland without discussing mating markets. When faced with a smaller mating pool (as minority Swedish-speakers arguably are) it might be a better strategy to hold on to the current partner rather than attempting to switch (Kokko & Jennions, 2008). Thus, based on a mating market perspective one might expect Swedish-speakers to separate *less* because they are fewer in number. Whether this is part of the explanation for lower divorce rates among Swedish-speakers is difficult to say. But based on this rationale, one might expect that separation should be especially low for Swedish-speakers in Finnish-speaking areas where they have fewer potential partners. Our data show, conversely, that separation rates of Swedish-speakers are actually somewhat *higher* in Finnish areas, not the reverse (as per *Hypothesis 3*, discussed above).

We refrain from making strong mating market claims in this study for two reasons. First, although Swedish-speakers often partner with one another, it is very common in Finland to partner across the language divide. It can therefore be argued that the potential mating pool of is the entire population, not just the Swedish-speakers. Second, Swedish-speakers who move to or live in Finnish-dominated areas may be different from their ethnolinguistic peers who reside in Swedish-dominated areas. The decision to move to another area may be taken just because that individual is perceiving mate scarcity. Interestingly, the number of people who have never had a cohabiting union at age 35, is higher for Finnish-speaking individuals, not Swedish-speaking individuals (Uggla & Saarela, 2024). This implies that a smaller relative number of partners does not necessarily hinder chances of ever having a stable union.

Negative child outcomes of divorce are well-documented across a range of societies, but the impact of divorce varies greatly. Evolutionary scholars contribute with an important insight: for an individual parent divorce can incur higher fitness benefits than staying in the pairbond. Whether that is the case depends both on the individual’s state and the local sociocultural context. In contemporary Finland, men and women with low incomes have more labile relationships and are more likely to remain childless (Jalovaara et al., 2019). Moreover, previous evidence from this population suggests that having a higher number of mates is associated with higher reproductive success (a positive Bateman gradient), but only for men with *low* incomes (Andersson, Jalovaara, Saarela & Uggla, 2023). For individuals with higher incomes, having multiple partners is not associated with higher lifetime fertility. However, cumulative number of years in a pairbond (another way to operationalize mating success) is positively correlated with higher reproductive success. The fitness benefits of deserting a mate to seek new mating opportunities may also differ between ethnic groups, norms in the local community and what type of social network individuals have access to. Future research could examine whether differences in fitness outcomes exist also across ethnolinguistic communities.

These data show that a two-generational approach to ethnolinguistic affiliation is necessary to understand separation risk and its transmission across generations, even in a contemporary welfare state where the two ethnic groups are similar on observable characteristics. For Finnish-speaking and Swedish-speaking couples alike, having divorced parents in Swedish areas where few others have divorced parents was associated with the highest risk of own separation. Future research could use child outcomes on, for example, education to examine whether the heterogeneous results shown here for separation also hold for other offspring outcomes earlier in life. Data on social support networks and survey data on divorce attitudes in these parallel communities could further help to disentangle the underlying mechanisms of intergenerational dynamics of separation.

## Supporting information

Uggla supplementary material Uggla supplementary material

## Supplementary material

The supplementary material for this article can be found at https://doi.org/10.1017/ehs.2025.9.

## Research transparency and reproducibility interest

The data used in this study are Finnish register data, which the authors cannot share. Code is available upon request.
