# Breaking-up and breaking the norm: intergenerational divorce transmission among two ethnolinguistic groups – CORRIGENDUM

**DOI:** 10.1017/ehs.2025.10004

**Published:** 2025-08-28

**Authors:** Caroline Uggla, Jan Saarela

In this Research Article the second author was missing.

The online Research Article has been corrected and the second author added.

We apologise for this error.
