# Supplementary material for: Breaking-up and breaking the norm: intergenerational divorce transmission among two ethnolinguistic groups
Source: Evol Hum Sci. 2025 Feb 18;7:e14. doi: 10.1017/ehs.2025.9 (PMC12034495; doi:10.1017/ehs.2025.9)
Supplement: Uggla supplementary material [file S2513843X2500009Xsup001.docx]

SUPPLEMENTARY MATERIAL.

**Breaking-up and breaking the norm:**

**Intergenerational divorce transmission among two ethnolinguistic groups**

|  | FF uniform | FF  mixed | SS uniform | SS  mixed | FS uniform | FS  mixed |
| --- | --- | --- | --- | --- | --- | --- |
| Number of couples | 497964 | 11745 | 10022 | 4812 | 9039 | 20755 |
| Number of couple years | 4388944 | 95232 | 110792 | 46570 | 76746 | 171813 |
| Number of separations | 279983 | 6953 | 3927 | 2266 | 4698 | 11849 |
| **Parental situation at age 17** |  |  |  |  |  |  |
| Both's parents' marriage intact | 58,2 | 49,2 | 73,1 | 65,3 | 60,8 | 54,2 |
| One's parents' marriage intact | 29,8 | 36,4 | 19,2 | 26,6 | 29,8 | 32,4 |
| No one's parents' marriage intact | 5,6 | 8,2 | 2,3 | 3,3 | 4,5 | 7,4 |
| Dead parent(s) | 6,4 | 6,2 | 5,4 | 4,8 | 4,9 | 6,0 |
| **Woman's age at union entry** |  |  |  |  |  |  |
| <=20 yrs | 24,8 | 21,7 | 19,2 | 16,6 | 14,4 | 20,2 |
| >=21 yrs & <=24 yrs | 35,3 | 35,2 | 39,6 | 42,0 | 32,3 | 35,6 |
| >=25 yrs & <=29 yrs | 26,1 | 26,9 | 29,5 | 30,1 | 31,7 | 28,5 |
| >=30 yrs | 13,9 | 16,1 | 11,7 | 11,2 | 21,7 | 15,7 |
| **Age difference man-woman** |  |  |  |  |  |  |
| <=-1 yrs | 19,9 | 20,7 | 18,5 | 18,8 | 23,8 | 20,8 |
| >=0 yrs & <=1 yrs | 34,6 | 34,2 | 33,1 | 36,7 | 31,4 | 34,7 |
| >=2 yrs & <=3 yrs | 24,9 | 24,1 | 27,2 | 25,8 | 24,1 | 23,7 |
| >=4 yrs | 20,6 | 20,9 | 21,2 | 18,7 | 20,7 | 20,8 |
| **Union entry year** |  |  |  |  |  |  |
| <=1999 | 33,8 | 30,7 | 34,8 | 32,0 | 30,0 | 31,0 |
| >=2000 & <=2004 | 31,4 | 32,7 | 31,8 | 32,1 | 29,9 | 31,1 |
| >=2005 & <=2009 | 23,2 | 23,8 | 24,2 | 25,5 | 25,0 | 24,7 |
| >=2010 | 11,6 | 12,8 | 9,2 | 10,4 | 15,1 | 13,2 |
| **Marital status at entry** |  |  |  |  |  |  |
| Cohabitation (nonmarried) | 90,6 | 93,6 | 87,5 | 92,2 | 93,5 | 93,4 |
| Marriage | 9,4 | 6,4 | 12,5 | 7,8 | 6,5 | 6,6 |
| **Woman's and man's union order** |  |  |  |  |  |  |
| Woman's first, man's first | 57,2 | 52,2 | 69,9 | 67,0 | 50,2 | 54,0 |
| Woman's 2+, man's first | 14,7 | 15,7 | 13,2 | 12,7 | 17,5 | 15,0 |
| 3 woman's first, man's 2+ | 12,3 | 13,3 | 9,8 | 11,5 | 13,9 | 14,0 |
| 4 woman's 2+, man's 2+ | 15,8 | 18,7 | 7,1 | 8,8 | 18,4 | 17,1 |
| **Number of children at entry** |  |  |  |  |  |  |
| No children | 81,9 | 81,5 | 83,7 | 85,0 | 80,6 | 82,2 |
| At least one common child, no children from previous union | 5,8 | 5,0 | 8,5 | 8,0 | 5,9 | 5,5 |
| Woman has previous children, man no previous children, with/without common children | 5,3 | 5,5 | 3,8 | 3,6 | 6,0 | 5,1 |
| Woman no previous children, man has previous children, with/without common children | 4,3 | 5,0 | 2,8 | 2,3 | 4,7 | 4,6 |
| Both have previous children, with/without common children | 2,7 | 3,0 | 1,3 | 1,1 | 2,7 | 2,6 |
| **Woman's education at entry** |  |  |  |  |  |  |
| Primary | 14,0 | 15,3 | 9,6 | 7,6 | 10,9 | 14,1 |
| Secondary, not matriculation examination | 23,1 | 19,2 | 26,6 | 19,8 | 17,2 | 17,9 |
| Secondary, matriculation examination | 34,4 | 38,1 | 26,4 | 34,7 | 33,7 | 38,4 |
| Tertiary | 28,5 | 27,4 | 37,4 | 37,9 | 38,2 | 29,5 |
| ***Continues on next page…*** |  |  |  |  |  |  |

| ***…continued from previous page*** |  |  |  |  |  |  |
| --- | --- | --- | --- | --- | --- | --- |
| **Man's education at entry** |  |  |  |  |  |  |
| Primary | 15,5 | 18,5 | 10,8 | 11,4 | 13,6 | 16,6 |
| Secondary, not matriculation examination | 37,7 | 31,5 | 43,0 | 30,8 | 29,9 | 28,2 |
| Secondary, matriculation examination | 25,8 | 30,0 | 18,6 | 29,2 | 27,7 | 32,5 |
| Tertiary | 20,9 | 20,1 | 27,5 | 28,6 | 28,8 | 22,7 |
| **Woman's religion at age 17** |  |  |  |  |  |  |
| Evangelical-Lutheran | 93,5 | 91,2 | 95,6 | 95,1 | 93,6 | 92,4 |
| No religion | 4,8 | 7,1 | 3,5 | 4,0 | 5,1 | 6,0 |
| Other religion | 1,7 | 1,7 | 0,9 | 0,9 | 1,3 | 1,6 |
| **Marital status at entry** |  |  |  |  |  |  |
| Cohabitation | 90,6 | 93,6 | 87,5 | 92,2 | 93,5 | 93,4 |
| Marriage | 9,4 | 6,4 | 12,5 | 7,8 | 6,5 | 6,6 |
| **Man's religion at age 17** |  |  |  |  |  |  |
| Evangelical-Lutheran | 93,0 | 91,2 | 95,0 | 94,1 | 93,0 | 92,2 |
| No religion | 5,4 | 7,0 | 4,0 | 4,7 | 5,6 | 6,3 |
| Other religion | 1,6 | 1,8 | 1,1 | 1,2 | 1,3 | 1,5 |
| **Woman's parents' education** |  |  |  |  |  |  |
| Both primary | 12,7 | 14,8 | 15,7 | 12,5 | 12,7 | 13,5 |
| One or both secondary, but none tertiary | 47,9 | 40,5 | 42,4 | 36,6 | 37,8 | 38,7 |
| One or both tertiary | 39,4 | 44,7 | 41,8 | 50,9 | 49,5 | 47,8 |
| **Man's parents' education** |  |  |  |  |  |  |
| Both primary | 14,0 | 15,0 | 17,5 | 13,2 | 14,8 | 14,6 |
| One or both secondary, but none tertiary | 46,3 | 40,4 | 39,8 | 35,5 | 35,1 | 37,7 |
| One or both tertiary | 39,6 | 44,6 | 42,7 | 51,3 | 50,2 | 47,7 |
| **Woman's full- and half-siblings** |  |  |  |  |  |  |
| Only full-sibling(s) | 73,7 | 66,3 | 83,7 | 77,7 | 74,6 | 70,3 |
| Only half-sibling(s) | 7,3 | 11,1 | 3,4 | 6,1 | 6,9 | 8,7 |
| Both full- and half-sibling(s) | 12,0 | 14,9 | 7,5 | 9,4 | 11,3 | 13,5 |
| No siblings | 7,0 | 7,7 | 5,4 | 6,8 | 7,2 | 7,5 |
| **Man's full- and half-siblings** |  |  |  |  |  |  |
| Only full-sibling(s) | 74,5 | 66,7 | 84,9 | 80,2 | 77,0 | 70,5 |
| Only half-sibling(s) | 6,8 | 10,8 | 3,2 | 4,9 | 5,6 | 8,2 |
| Both full- and half-sibling(s) | 11,1 | 14,4 | 6,8 | 8,4 | 10,2 | 13,2 |
| No siblings | 7,5 | 8,1 | 5,1 | 6,5 | 7,3 | 8,2 |
| **Population density at entry** |  |  |  |  |  |  |
| Urban | 76,7 | 86,9 | 49,6 | 65,1 | 79,9 | 85,2 |
| Semi-urban | 11,7 | 7,9 | 19,1 | 16,3 | 11,6 | 8,4 |
| Rural | 11,6 | 5,2 | 31,3 | 18,6 | 8,5 | 6,4 |
| **Proportion Swedish speakers in municipality at entry** |  |  |  |  |  |  |
| <0.004 | 47,7 | 11,4 | 0,1 | 0,1 | 2,5 | 8,3 |
| >=0.004 & <0.015 | 20,5 | 12,5 | 0,2 | 0,4 | 5,2 | 10,2 |
| >=0.015 & <0.100 | 25,6 | 50,2 | 15,3 | 30,5 | 45,4 | 49,3 |
| >=0.100 & <0.300 | 5,3 | 18,8 | 15,3 | 19,0 | 20,8 | 18,8 |
| >=0.300 & <0.500 | 0,7 | 4,4 | 7,8 | 10,7 | 11,6 | 6,4 |
| >=0.500 & <0.750 | 0,2 | 2,3 | 19,1 | 17,1 | 8,7 | 4,2 |
| >=0.750 | 0,0 | 0,4 | 42,3 | 22,2 | 5,9 | 2,8 |

**Table A1.** Number of couples, couple years and separations, and percentage distribution of independent variables. Percentage distribution by couple’s ethnolinguistic category FF uniform (both partners registered as Finnish-speaking, and their two sets of parents also all Finnish-registered). FF mixed- both partners registered as Finnish-speaking but at least one of their parents are Swedish-registered, SS uniform (both partners registered as Swedish-speaking, and their two sets of parents also all Swedish-registered), SS mixed (both partners Swedish-registered, but at least one parent Finnish-registered), FS uniform (one partner is Finnish-registered and has two Finnish-registered parents, the other partner is Swedish-registered and has two Swedish-registered parents, FS mixed (one partner is Swedish-registered and the other is Finnish-registered, and at least one of the partners come have parents who have mixed backgrounds).

|  |  | Hazard ratio (HR) | p-value | 95% CI low | 95% CI high |
| --- | --- | --- | --- | --- | --- |
| **F_F uniform F** | **Both parents’ marriage intact** | **1** |  |  |  |
|  | One's parents' marriage intact | 1,18 | 0,000 | 1,17 | 1,19 |
|  | No one's parents' marriage intact | 1,30 | 0,000 | 1,28 | 1,32 |
|  | Dead parent(s) | 1,10 | 0,000 | 1,09 | 1,12 |
| **F_F mixed background** | Both's parents' marriage intact | 1,02 | 0,300 | 0,98 | 1,06 |
|  | One's parents' marriage intact | 1,19 | 0,000 | 1,15 | 1,23 |
|  | No one's parents' marriage intact | 1,33 | 0,000 | 1,24 | 1,42 |
|  | Dead parent(s) | 1,16 | 0,002 | 1,05 | 1,27 |
| **S_S uniform background** | Both's parents' marriage intact | 0,76 | 0,000 | 0,72 | 0,79 |
|  | One's parents' marriage intact | 0,98 | 0,629 | 0,92 | 1,05 |
|  | No one's parents' marriage intact | 1,13 | 0,098 | 0,98 | 1,30 |
|  | Dead parent(s) | 0,85 | 0,024 | 0,75 | 0,98 |
| **S_S mixed background** | Both's parents' marriage intact | 0,89 | 0,000 | 0,83 | 0,94 |
|  | One's parents' marriage intact | 1,14 | 0,000 | 1,06 | 1,23 |
|  | No one's parents' marriage intact | 1,37 | 0,000 | 1,17 | 1,62 |
|  | Dead parent(s) | 1,13 | 0,170 | 0,95 | 1,34 |
| **S_F uniform S/F background** | Both's parents' marriage intact | 1,05 | 0,024 | 1,01 | 1,09 |
|  | One's parents' marriage intact | 1,18 | 0,000 | 1,12 | 1,23 |
|  | No one's parents' marriage intact | 1,27 | 0,000 | 1,15 | 1,41 |
|  | Dead parent(s) | 1,16 | 0,021 | 1,02 | 1,31 |
| **S_F mixed background** | Both's parents' marriage intact | 1,05 | 0,000 | 1,03 | 1,08 |
|  | One's parents' marriage intact | 1,20 | 0,000 | 1,16 | 1,23 |
|  | No one's parents' marriage intact | 1,26 | 0,000 | 1,20 | 1,33 |
|  | Dead parent(s) | 1,06 | 0,116 | 0,99 | 1,15 |
| **Woman's age at union entry** | <=20 yrs | 1 |  |  |  |
|  | >=21 yrs & <=24 yrs | 0,81 | 0,000 | 0,80 | 0,82 |
|  | >=25 yrs & <=29 yrs | 0,67 | 0,000 | 0,67 | 0,68 |
|  | >=30 yrs | 0,58 | 0,000 | 0,57 | 0,59 |
| **Age difference**  **man-woman** | <=-1 yrs | 1 |  |  |  |
|  | >=0 yrs & <=1 yrs | 0,88 | 0,000 | 0,87 | 0,89 |
|  | >=2 yrs & <=3 yrs | 0,88 | 0,000 | 0,87 | 0,89 |
|  | >=4 yrs | 0,91 | 0,000 | 0,90 | 0,92 |
| **Union entry year** | <=1999 |  |  |  |  |
|  | >=2000 & <=2004 | 1,01 | 0,214 | 1,00 | 1,02 |
|  | >=2005 & <=2009 | 0,98 | 0,005 | 0,97 | 1,00 |
|  | >=2010 | 0,93 | 0,000 | 0,91 | 0,94 |
| **Marital status at entry** | Cohabitation (nonmarried) | 1 |  |  |  |
|  | Marriage | 0,52 | 0,000 | 0,51 | 0,53 |
| **Woman's and man's union order** | Woman's first, man's first |  |  |  |  |
|  | Woman's 2+, man's first | 1,25 | 0,000 | 1,23 | 1,26 |
|  | 3 woman's first, man's 2+ | 1,16 | 0,000 | 1,15 | 1,18 |
|  | 4 woman's 2+, man's 2+ | 1,30 | 0,000 | 1,28 | 1,32 |
| ***Continues on next page*** |  |  |  |  |  |

| ***Continued.*** |  |  |  |  |  |
| --- | --- | --- | --- | --- | --- |
| **Number of children at entry** | No children |  |  |  |  |
|  | At least one common child, no children from previous union | 0,87 | 0,000 | 0,85 | 0,88 |
|  | Woman has previous children, man no previous children, with/without common children | 1,10 | 0,000 | 1,08 | 1,12 |
|  | Woman no previous children, man has previous children, with/without common children | 1,13 | 0,000 | 1,11 | 1,15 |
|  | Both have previous children, with/without common children | 1,31 | 0,000 | 1,28 | 1,34 |
| **Woman's education at entry** | Primary | 1 |  |  |  |
|  | Secondary, not matriculation examination | 0,81 | 0,000 | 0,80 | 0,82 |
|  | Secondary, matriculation examination | 0,82 | 0,000 | 0,81 | 0,83 |
|  | Tertiary | 0,62 | 0,000 | 0,62 | 0,63 |
| **Man's education at entry** | Primary |  |  |  |  |
|  | Secondary, not matriculation examination | 0,80 | 0,000 | 0,79 | 0,81 |
|  | Secondary, matriculation examination | 0,81 | 0,000 | 0,80 | 0,82 |
|  | Tertiary | 0,63 | 0,000 | 0,62 | 0,64 |
| **Woman's religion at age 17** | Evangelical-Lutheran |  |  |  |  |
|  | No religion | 1,10 | 0,000 | 1,09 | 1,12 |
|  | Other religion | 1,02 | 0,129 | 0,99 | 1,05 |
| **Man's religion at age 17** | Evangelical-Lutheran | 1 |  |  |  |
|  | No religion | 1,04 | 0,000 | 1,02 | 1,05 |
|  | Other religion | 0,98 | 0,170 | 0,95 | 1,01 |
| **Woman's parents' education** | Both primary | 1 |  |  |  |
|  | One or both secondary, but none tertiary | 1,05 | 0,000 | 1,04 | 1,06 |
|  | One or both tertiary | 1,13 | 0,000 | 1,11 | 1,14 |
| **Man's parents' education** | Both primary | 1 |  |  |  |
|  | One or both secondary, but none tertiary | 1,03 | 0,000 | 1,02 | 1,04 |
|  | One or both tertiary | 1,08 | 0,000 | 1,06 | 1,09 |
| **Woman's full- and half-siblings** | Only full-sibling(s) | 1 |  |  |  |
|  | Only half-sibling(s) | 1,09 | 0,000 | 1,08 | 1,11 |
|  | Both full- and half-sibling(s) | 1,09 | 0,000 | 1,08 | 1,11 |
|  | No siblings | 1,03 | 0,000 | 1,01 | 1,04 |
| **Man's full- and half-siblings** | Only full-sibling(s) | 1 |  |  |  |
|  | Only half-sibling(s) | 1,10 | 0,000 | 1,08 | 1,11 |
|  | Both full- and half-sibling(s) | 1,07 | 0,000 | 1,06 | 1,09 |
|  | No siblings | 1,02 | 0,001 | 1,01 | 1,04 |
| **Population density at entry** | Urban | 1 |  |  |  |
|  | Semi-urban | 0,91 | 0,000 | 0,90 | 0,93 |
|  | Rural | 0,86 | 0,000 | 0,85 | 0,87 |
| **Proportion Swedish speakers in municipality at entry** | <0.004 | 1 |  |  |  |
|  | >=0.004 & <0.015 | 1,00 | 0,671 | 0,99 | 1,01 |
|  | >=0.015 & <0.100 | 1,06 | 0,000 | 1,05 | 1,07 |
|  | >=0.100 & <0.300 | 0,96 | 0,000 | 0,94 | 0,97 |
|  | >=0.300 & <0.500 | 1,00 | 0,823 | 0,97 | 1,03 |
|  | >=0.500 & <0.750 | 0,92 | 0,000 | 0,88 | 0,96 |
|  | >=0.750 | 0,94 | 0,013 | 0,90 | 0,99 |
| **Table A2**. Event history model (hazard ratios) of separation/divorce of focal couple (full model with all  controls). | | | | | |

| Couple’s ethno-  linguistic  cateory | Couple’s parents divorce | Ethno-  linguistic profile of municipality | Hazard ratio (HR) | p-value | 95% CI low | 95% CI high |
| --- | --- | --- | --- | --- | --- | --- |
| FF | Both's parents' marriage intact | Finnish | **REF** |  |  |  |
| FF | One's parents' marriage intact | Finnish | 1,18 | 0,000 | 1,17 | 1,19 |
| FF | No one's parents' marriage intact | Finnish | 1,30 | 0,000 | 1,28 | 1,32 |
| FF | Dead parent(s) | Finnish | 1,10 | 0,000 | 1,09 | 1,12 |
| SS | Both's parents' marriage intact | Finnish | 0,83 | 0,000 | 0,79 | 0,87 |
| SS | One's parents' marriage intact | Finnish | 1,00 | 0,929 | 0,94 | 1,07 |
| SS | No one's parents' marriage intact | Finnish | 1,21 | 0,021 | 1,03 | 1,42 |
| SS | Dead parent(s) | Finnish | 1,06 | 0,450 | 0,91 | 1,23 |
| FS | Both's parents' marriage intact | Finnish | 1,07 | 0,000 | 1,04 | 1,10 |
| FS | One's parents' marriage intact | Finnish | 1,22 | 0,000 | 1,19 | 1,26 |
| FS | No one's parents' marriage intact | Finnish | 1,32 | 0,000 | 1,24 | 1,40 |
| FS | Dead parent(s) | Finnish | 1,14 | 0,001 | 1,05 | 1,23 |
| FF | Both's parents' marriage intact | Swedish | 0,94 | 0,242 | 0,85 | 1,04 |
| FF | One's parents' marriage intact | Swedish | 1,10 | 0,071 | 0,99 | 1,21 |
| FF | No one's parents' marriage intact | Swedish | 1,27 | 0,016 | 1,04 | 1,53 |
| FF | Dead parent(s) | Swedish | 1,04 | 0,753 | 0,80 | 1,36 |
| SS | Both's parents' marriage intact | Swedish | 0,71 | 0,000 | 0,68 | 0,75 |
| SS | One's parents' marriage intact | Swedish | 1,00 | 0,969 | 0,94 | 1,07 |
| SS | No one's parents' marriage intact | Swedish | 1,13 | 0,067 | 0,99 | 1,29 |
| SS | Dead parent(s) | Swedish | 0,75 | 0,000 | 0,65 | 0,87 |
| FS | Both's parents' marriage intact | Swedish | 0,97 | 0,535 | 0,89 | 1,06 |
| FS | One's parents' marriage intact | Swedish | 1,09 | 0,078 | 0,99 | 1,20 |
| FS | No one's parents' marriage intact | Swedish | 1,22 | 0,029 | 1,02 | 1,45 |
| FS | Dead parent(s) | Swedish | 0,99 | 0,941 | 0,78 | 1,26 |

**Table A3a.** Event history model for separation/divorce of the focal couple. Reference category FF couple, in Finnish-area with both parent’s marriages intact. Controlling for woman’s age at union entry, age difference man-woman in years, year of union entry, marital status at entry, woman’s and man’s union order, number of children at union entry, woman’s education at union entry, man’s education at union entry, woman’s religion at age 17 years, man’s religion at age 17 years, woman’s parents’ education, man’s parents’ education, woman’s full- and halfsiblings, man’s full- and half-siblings, population density at entry (for more details of independent variable categories, see methods section).

| Couple’s ethno-  linguistic  cateory | Couple’s parents divorce | Ethno-  linguistic profile of municipality | Hazard ratio (HR) | p-value | 95% CI low | 95% CI high |
| --- | --- | --- | --- | --- | --- | --- |
| FF | Both's parents' marriage intact | Finnish | 1,21 | 0,000 | 1,16 | 1,26 |
| FF | One's parents' marriage intact | Finnish | 1,42 | 0,000 | 1,36 | 1,49 |
| FF | No one's parents' marriage intact | Finnish | 1,57 | 0,000 | 1,50 | 1,65 |
| FF | Dead parent(s) | Finnish | 1,33 | 0,000 | 1,27 | 1,40 |
| SS | Both's parents' marriage intact | Finnish | **REF** |  |  |  |
| SS | One's parents' marriage intact | Finnish | 1,21 | 0,000 | 1,12 | 1,31 |
| SS | No one's parents' marriage intact | Finnish | 1,46 | 0,000 | 1,23 | 1,72 |
| SS | Dead parent(s) | Finnish | 1,28 | 0,002 | 1,10 | 1,49 |
| FS | Both's parents' marriage intact | Finnish | 1,29 | 0,000 | 1,23 | 1,36 |
| FS | One's parents' marriage intact | Finnish | 1,48 | 0,000 | 1,40 | 1,56 |
| FS | No one's parents' marriage intact | Finnish | 1,59 | 0,000 | 1,48 | 1,72 |
| FS | Dead parent(s) | Finnish | 1,38 | 0,000 | 1,26 | 1,51 |
| FF | Both's parents' marriage intact | Swedish | 1,14 | 0,023 | 1,02 | 1,27 |
| FF | One's parents' marriage intact | Swedish | 1,33 | 0,000 | 1,19 | 1,48 |
| FF | No one's parents' marriage intact | Swedish | 1,53 | 0,000 | 1,26 | 1,86 |
| FF | Dead parent(s) | Swedish | 1,26 | 0,089 | 0,97 | 1,64 |
| SS | Both's parents' marriage intact | Swedish | 0,86 | 0,000 | 0,81 | 0,92 |
| SS | One's parents' marriage intact | Swedish | 1,21 | 0,000 | 1,12 | 1,31 |
| SS | No one's parents' marriage intact | Swedish | 1,36 | 0,000 | 1,19 | 1,56 |
| SS | Dead parent(s) | Swedish | 0,91 | 0,226 | 0,78 | 1,06 |
| FS | Both's parents' marriage intact | Swedish | 1,18 | 0,001 | 1,07 | 1,29 |
| FS | One's parents' marriage intact | Swedish | 1,32 | 0,000 | 1,18 | 1,46 |
| FS | No one's parents' marriage intact | Swedish | 1,47 | 0,000 | 1,23 | 1,77 |
| FS | Dead parent(s) | Swedish | 1,20 | 0,148 | 0,94 | 1,53 |

Table A3b. Event history model for separation/divorce of the focal couple. Reference category FF couple, in Finnish-area with both parent’s marriages intact. Controlling for woman’s age at union entry, age difference man-woman in years, year of union entry, marital status at entry, woman’s and man’s union order, number of children at union entry, woman’s education at union entry, man’s education at union entry, woman’s religion at age 17 years, man’s religion at age 17 years, woman’s parents’ education, man’s parents’ education, woman’s full- and halfsiblings, man’s full- and half-siblings, population density at entry (for more details of independent variable categories, see methods section).

| Couple’s ethno-  linguistic  cateory | Couple’s parents divorce | Ethno-  linguistic profile of municipality | Odds ratio (OR) | p-value | 95% CI low | 95% CI high |
| --- | --- | --- | --- | --- | --- | --- |
| FF | Both's parents' marriage intact | Finnish | 0,93 | 0,000 | 0,91 | 0,96 |
| FF | One's parents' marriage intact | Finnish | 1,10 | 0,000 | 1,07 | 1,13 |
| FF | No one's parents' marriage intact | Finnish | 1,22 | 0,000 | 1,18 | 1,25 |
| FF | Dead parent(s) | Finnish | 1,03 | 0,050 | 1,00 | 1,06 |
| SS | Both's parents' marriage intact | Finnish | 0,77 | 0,000 | 0,73 | 0,81 |
| SS | One's parents' marriage intact | Finnish | 0,94 | 0,057 | 0,88 | 1,00 |
| SS | No one's parents' marriage intact | Finnish | 1,13 | 0,149 | 0,96 | 1,33 |
| SS | Dead parent(s) | Finnish | 0,99 | 0,878 | 0,85 | 1,15 |
| FS | Both's parents' marriage intact | Finnish | **REF** |  |  |  |
| FS | One's parents' marriage intact | Finnish | 1,14 | 0,000 | 1,10 | 1,19 |
| FS | No one's parents' marriage intact | Finnish | 1,23 | 0,000 | 1,15 | 1,31 |
| FS | Dead parent(s) | Finnish | 1,07 | 0,137 | 0,98 | 1,16 |
| FF | Both's parents' marriage intact | Swedish | 0,88 | 0,016 | 0,79 | 0,98 |
| FF | One's parents' marriage intact | Swedish | 1,03 | 0,642 | 0,92 | 1,14 |
| FF | No one's parents' marriage intact | Swedish | 1,18 | 0,090 | 0,97 | 1,43 |
| FF | Dead parent(s) | Swedish | 0,97 | 0,845 | 0,75 | 1,27 |
| SS | Both's parents' marriage intact | Swedish | 0,67 | 0,000 | 0,63 | 0,70 |
| SS | One's parents' marriage intact | Swedish | 0,94 | 0,052 | 0,87 | 1,00 |
| SS | No one's parents' marriage intact | Swedish | 1,05 | 0,432 | 0,92 | 1,20 |
| SS | Dead parent(s) | Swedish | 0,70 | 0,000 | 0,60 | 0,82 |
| FS | Both's parents' marriage intact | Swedish | 0,91 | 0,036 | 0,83 | 0,99 |
| FS | One's parents' marriage intact | Swedish | 1,02 | 0,736 | 0,92 | 1,12 |
| FS | No one's parents' marriage intact | Swedish | 1,14 | 0,158 | 0,95 | 1,36 |
| FS | Dead parent(s) | Swedish | 0,93 | 0,529 | 0,73 | 1,18 |

**Table A3c**. Event history model for separation/divorce of the focal couple. Reference category FF couple, in Finnish-area with both parent’s marriages intact. Controlling for woman’s age at union entry, age difference man-woman in years, year of union entry, marital status at entry, woman’s and man’s union order, number of children at union entry, woman’s education at union entry, man’s education at union entry, woman’s religion at age 17 years, man’s religion at age 17 years, woman’s parents’ education, man’s parents’ education, woman’s full- and halfsiblings, man’s full- and half-siblings, population density at entry (for more details of independent variable categories, see methods section).
